# Case of Ununited Fracture of the Arm, Successfully Treated by Pressure

**Published:** 1827-07

**Authors:** 

**Affiliations:** St. George's Hospital


### MISCELLANEOUS CASES.

*Case of Ununited Fracture of the Arm, successfully treated by Pressure, at* ST. GEORGE'S HOSPITAL, *by* MR. BRODIE.

JAMES M'EWEN, twenty-four years of age, in November, 1825, met with an accident, which occasioned a fracture of the right arm and the left leg. He was taken to a public hospital, where the fractures were treated in the usual manner. The fracture of the leg became united, but no union took place of the fracture of the arm. Sometime in August, 1826, he was admitted into a charitable institution, the surgeon of which passed a seton through the arm, between the fractured extremities of the bone. The seton was withdrawn at the expiration of a week. Some inflammation causing the surrounding soft parts to become consolidated and thickened, must have followed the operation, as it was supposed that a cure had been effected.† When, however, the inflammation had subsided, it was found that the bones were as loose and moveable as ever.

On the 29th of November, 1826, the man was admitted into St. George's Hospital. At this time the broken ends of the bone appeared to be united by ligament, admitting of extensive motion. They rode considerably one over the other, and the limb was

† See Lancet, vol. xi. p. 264.

somewhat shortened. The following plan of treatment was adopted, founded on a principle suggested by Mr. Amesbury.

The forearm being placed in the half-bent position, a wooden splint, adapted to the half-bent figure of the limb, was applied on the inside, extending from the axilla to the fingers; a straight splint was then placed on the outside of the arm, extending from the shoulder to the outer condyle; and these splints were secured by bandages. On the outside of all, a tourniquet was applied, the band of which embraced the limb in the situation of the fracture. By turning the screw, which was on the outside of the arm, additional pressure was made on the fractured bones; and it was easy to regulate the pressure, so that it should be as great as possible without occasioning much distress to the patient. The curved wooden splint in the inside being broader than the limb, and only slightly concave, the principal blood-vessels were defended from pressure, so that, whatever force was employed for the purpose of keeping the fractured ends of the bones in close contact, the circulation of the limb continued nearly uninterrupted.

This plan having been pursued for about six weeks, it was found that the fractured bones admitted of much less motion than before. At the end of three months, no motion whatever was perceptible; but, by way of security, the use of the apparatus was continued for another month.

On the 31st of May, the man left the hospital, having been for some time allowed the free exercise of the limb, without splints or bandages, the bones being firmly consolidated, and the limb as useful as it was before the accident.

